# Deep sequencing analysis of transcriptomes in *Aspergillus flavus* in response to resveratrol

**DOI:** 10.1186/s12866-015-0513-6

**Published:** 2015-09-16

**Authors:** Houmiao Wang, Yong Lei, Liying Yan, Ke Cheng, Xiaofeng Dai, Liyun Wan, Wei Guo, Liangqiang Cheng, Boshou Liao

**Affiliations:** Key Laboratory of Oil Crop Biology of the Ministry of Agriculture, Oil Crops Research Institute of Chinese Academy of Agricultural Sciences, Wuhan, 430062 China; Chinese Academy of Agricultural Sciences–International Crop Research Institute for the Semi-Arid Tropics Joint Laboratory for Groundnut Aflatoxin Management, Wuhan, 430062 China; Institute of Agro-Products Processing Science and Technology, Chinese Academy of Agricultural Sciences, Beijing, 100193 China

## Abstract

**Background:**

Resveratrol has been reported as a natural phytoalexin that inhibits infection or the growth of certain fungi including *Aspergillus flavus*. Our previous research revealed that aflatoxin production in *A. flavus* was reduced in medium with resveratrol. To understand the molecular mechanism of the *A. flavus* response to resveratrol treatment, the high-throughput paired-end RNA-Seq was applied to analyze the transcriptomic profiles of *A. flavus*.

**Results:**

In total, 366 and 87 genes of *A. flavus* were significantly up- and down- regulated, respectively, when the fungus was treated with resveratrol. Gene Ontology (GO) functional enrichment analysis revealed that 48 significantly differentially expressed genes were involved in 6 different terms. Most genes in the aflatoxin biosynthetic pathway genes cluster (#54) did not show a significant change when *A. flavus* was treated with resveratrol, but 23 of the 30 genes in the #54 cluster were down-regulated. The transcription of *aflA* and *aflB* was significantly suppressed under resveratrol treatment, resulting in an insufficient amount of the starter unit hexanoate for aflatoxin biosynthesis. In addition, resveratrol significantly increased the activity of antioxidative enzymes that destroy radicals, leading to decreased aflatoxin production. Moreover, *stuA*, *fluG*, *flbC*, and others genes involved in mycelial and conidial development were down-regulated, which disrupted the cell’s orderly differentiation and blocked conidia formation and mycelia development. The transcripts of *laeA* and *veA* were slightly inhibited by resveratrol, which may partly decrease aflatoxin production and depress conidia formation.

**Conclusions:**

Resveratrol can affect the expression of *A. flavus* genes that are related to developmental and secondary metabolic processes, resulting in decreased aflatoxin production and conidia formation and could also cause abnormal mycelia development. These results provide insight into the transcriptome of *A. flavus* in response to resveratrol and a new clew for further study in regulation of aflatoxin biosynthesis in *A. flavus*.

**Electronic supplementary material:**

The online version of this article (doi:10.1186/s12866-015-0513-6) contains supplementary material, which is available to authorized users.

## Background

The widespread fungus *Aspergillus flavus* is a saprophytic and opportunistic pathogen that is capable of causing diseases in agricultural crops [1], producing toxic secondary metabolites, and bringing about health problems in animals and humans [2]. Under favorable conditions, *A. flavus* produces polyketide-derived carcinogenic and mutagenic secondary metabolites, aflatoxins [3], which are extremely hepatotoxic, immunosuppressive, and antinutritional mycotoxins associated with both acute and chronic toxicity in humans and animals [4]. Besides the health implications in humans and animals, *A. flavus* colonization in crops causes considerable economic losses because of reduced utilization and lower price of aflatoxin-contaminated grains.

To develop effective means of combating aflatoxin contamination, it is of vital importance to investigate the molecular mechanisms of development and secondary metabolism in *A. flavus*. Previous studies identified numerous diverse biochemical substances and extracts with adverse effects on the development and/or secondary metabolism of *A. flavus* [5–10]. Resveratrol (3,5,4-trihydroxystilbene) is a phytoalexin with antifungal activity and antioxidant capacity, which can enhance host plant resistance against biotic and abiotic stresses [11]. Our previous study found that resveratrol is highly related to resistance to aflatoxin production in peanut seeds and could inhibit aflatoxin production in medium [12]. In addition, resveratrol affected mycelial morphologic characteristics and inhibited conidia formation of *A. flavus* in medium. However, how resveratrol affects the development and secondary metabolism of *A. flavus* remains unknown.

RNA-sequencing (RNA-seq) is a powerful and cost-efficient high-throughput technology for transcriptomic profiling analysis that has been successfully used to interrogate transcriptomes of numerous fungi [10, 13–17]. The application of this technology has greatly accelerated our understanding of the complexity of gene expression, regulation, and networks [18]. Gene expression can be more accurately quantified using RNA-seq approaches than conventional transcriptomic analysis [18]. With higher sensitivity, RNA-seq can also be more efficient in detecting a larger range of dynamically expressed genes than microarrays. Furthermore, RNA-seq has been used to survey sequence variations and complex transcriptomes with low false-positive rates and high sensitivity and reproducibility [14, 19]. For an organism with a well-annotated genome, mapping read sequences to the corresponding reference genome/transcriptome is usually the first and essential step for RNA-seq data analysis [10]. Moreover, the “reference-based” RNA-seq approach was proved to be more sensitive and have higher computational efficiency than a *de novo* assembly approach [20]. The construction of expressed sequences tags in *A. flavus* was reported by several researchers [21], and the whole-genome sequencing of the fungus was completed [22]. Annotation of the *A. flavus* genome revealed numerous genes and gene clusters that are potentially involved in conidia formation and secondary metabolic processes including aflatoxin biosynthesis [23, 24]. The transcriptomic analysis of aflatoxin biosynthesis and mycelia development in *A. flavus* in response to 5-azacytidine [10, 25], decanal [17], water activity [26], and temperature [27] also used RNA-seq technology.

To comprehensively understand the effect of resveratrol on the development and secondary metabolism of *A. flavus*, an RNA-seq approach was applied in this study to obtain transcriptomic profiles of *A. flavus* at the whole-genome level. Differentially expressed genes (DEGs) associated with mycelial growth, conidia formation, and aflatoxin biosynthesis were revealed by comparing resveratrol-treated and untreated samples of *A. flavus*. This study would be meaningful for further annotating the genome of *A. flavus*, an understanding of aflatoxin biosynthesis and improving aflatoxin contamination management in agricultural products.

## Results

### Effect of resveratrol on the development and aflatoxin production of *A. flavus*

**Fig. 1:**
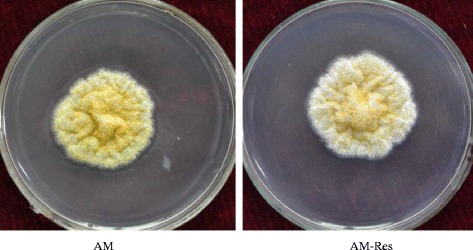
Mycelioid colony of *A. flavus* cultured on solid A & M medium. AM (Control), resveratrol was not added to the A&M medium. AM-Res (Treatment), resveratrol was added to the A&M medium (3.0 μg/mL). The mycelioid colony diameter of *A. flavus* in the AM-Res was similar to that in the AM, but the number of *A. flavus* conidia in the AM was greater than that in the AM-Res

**Fig. 2:**
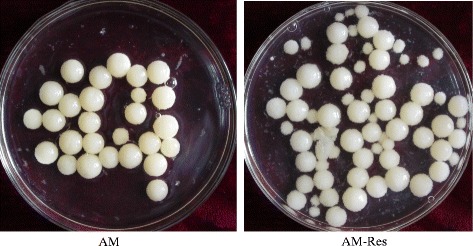
Vegetative mycelia of *A. flavus* cultured in liquid A & M medium. AM (Control), resveratrol was not added to the A&M medium. AM-Res (Treatment), resveratol was added to the A&M medium (3.0 μg/mL)

Morphological characteristics and aflatoxin concentrations were quantified to define the effect of resveratrol on the fungal development and toxigenicity of *A. flavus*. The dry biomass of *A. flavus* mycelia grown in liquid A&M medium did not show significant difference between the resveratrol treatment (AM-Res) and the control (AM) (Table 1). A similar result was obtained using solid A&M medium, in which resveratrol treatment did not significantly change the diameter of fungal mycelioid colonies (Fig. 1 and Table 1). However, obvious variation in morphological characteristics of mycelia in response to resveratrol treatment in shaking liquid culture was observed (Fig. 2). The vegetative mycelial morphologic characteristics of *A. flavus* were different between the AM and the AM-Res cultures, and the vegetative mycelia of *A. flavus* appeared some smaller ones in the AM-Res. Resveratrol may affect the germination rate of conidia or growth rate of vegetative mycelia, leading to differences in the length of the mycelia. The results indicated that resveratrol did not affect the biomass accumulation of *A. flavus*, but it could influence the development of fungal mycelia.

**Table 1:** Effect of resveratrol on the development and aflatoxin production of *A. flavus*

	Mycelia biomass	AF production	Mycelioid colony diameter	Conidia number
	(g/60 mL)	(μg/L)	(cm)	(1 × 10^6^ CFU/20 mL)
AM (Control)	0.411 ± 0.027	385.49 ± 17.38	7.1 ± 0.27	285.31 ± 52.50
AM-Res (Treatment)	0.464 ± 0.0067	203.55 ± 31.46 *	7.6 ± 0.35	175.21 ± 40.17 *

Note: AM-Res (Treatment) and AM (Control) indicate that *A. flavus* was cultured in A&M medium with or without resveratrol, respectively. * denotes significant differences (*p* < 0.05) between mean values of AM-Res and AM by a *t*-test

Because no fungal conidium was detected in liquid culture, solid A&M medium was used to investigate the effect of resveratrol on conidia production by *A. flavus*. The number of conidia was 285.31 × 10^6^ and 175.21 × 10^6^ per 20 mL medium in the control and resveratrol treatment (3.0 μg/mL), respectively (Fig. 1 and Table 1). In addition, the number of conidia was 197.83 × 10^6^, 180.83 × 10^6^, 163.06 × 10^6^, and 164.17 × 10^6^ per 20 mL medium with resveratrol at concentrations of 0.3, 15.0, 30.0, and 60.0 μg/mL, respectively. Conidia formation of *A. flavus* was decreased in the resveratrol treatment compared with the control.

The aflatoxin concentration was measured after 7 days of growth in liquid culture. The aflatoxin content in the resveratrol-treated culture (3.0 μg/mL) was 203.6 μg/L, which was much less than that in the control (Table 1), indicating that resveratrol inhibited aflatoxin formation. Our previous results also indicated that a resveratrol concentration of 3.0 μg/mL or higher inhibited aflatoxin production [12].

### Defining the *A. flavus* transcriptome

**Fig. 3:**
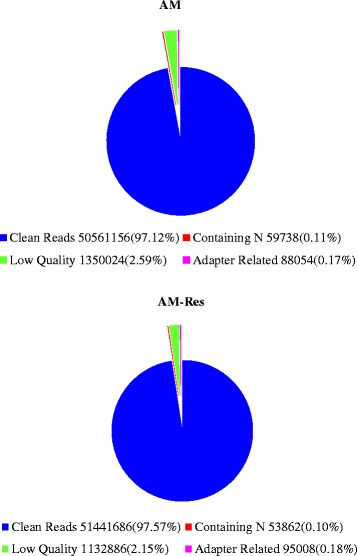
Classification of raw reads. Raw reads including clean reads (blue), adapter sequences (purple), reads containing undefined nucleotides (N’s) (red), and low-quality reads (green) generated from Illumina RNA-sequencing (RNA-seq)

**Fig. 4:**
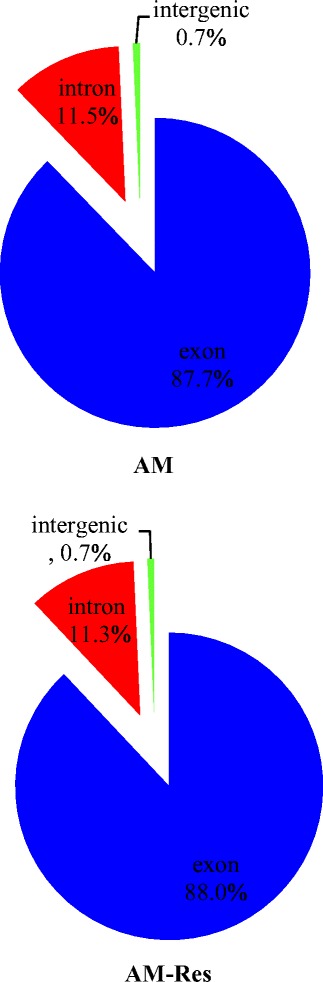
Distribution of reads mapped to genome regions. The genic distribution of reads from mRNA-seq of *A. flavus* (AF2202) mapped to exons (blue), introns (red), and intergenic regions (green)

Illumina sequencing of the resveratrol-treated and untreated *A. flavus* AF2202 generated 52.06 million and 52.72 million raw reads per library, respectively. Among total reads, approximately 51 million reads were purified from each group (Fig. 3), which included 46.00 million (89.42 %, AM-Res) and 45.34 million (89.68 %, AM) reads that were uniquely mapped to the *A. flavus* reference genome (Additional file 1). The data sets could represent the expressed sequences or transcriptomes of each library. Matching the reads to genes is important to annotate sequences because it could reveal the molecular mechanism behind gene expression [28]. Thus, transcriptomic data obtained in this experiment were used not only to define the differential gene expression in response to resveratrol but also to identify hitherto unknown transcripts including putative isoforms. The genic distribution of reads from mRNA-seq of the resveratrol-treated AF2202 (AM-Res) indicated that most reads (88.0 %) were mapped to protein-coding genes. The others were distributed between introns (0.7 %) and intergenic regions (11.3 %) (Fig. 4). The distribution of reads in the control AF2202 (AM) was similar to that of the resveratrol-treated (AM-Res), but fewer reads in the control were mapped to exons (Fig. 4). In total, 10.2 Gb of valid clean reads were yielded, and 98.82 % (9.13 Gb) of the 9.24 Gb of collected read pairs that passed filtering were mapped uniquely to the genome (Additional file 1), which was higher than the RNA-seq data published previously for *A. flavus* [10, 26, 27] and *A. oryzae* [13].

In 2005, a 5× sequence coverage of the *A. flavus* genome was released by J. Craig Venter Research Institute, which has been recently updated at several web sites (http://www.aspergillusflavus.org/genomics/, NCBI). The *A. flavus* genome is about 37 Mb on 8 chromosomes, and it encodes over 13,487 functional genes (*Aspergillus flavus* Genome Sequencing Project), which previously were annotated as open reading frames in the genome without unambiguous transcript information. We quantified the overall transcriptional activity of genes in this study by calculating the number of reads per kilobase of exon per million mapped reads (RPKM) [29]. We found that 9,870 (71.14 %) of the 13,875 genes (http://fungi.ensembl.org/Aspergillus_flavus/Info/Annotation/#assembly) in the *A. flavus* genome database had expression with RPKM ≥ 1 in either of the two samples (Additional files 2 and 3).

Besides the small fraction of reads that mapped to rRNA, approximately 11 % of reads could not be mapped to the encoding regions. This might be due to non-coding RNAs or novel exons of known genes. Novel genes were identified by assembling the RNA-seq data from the two samples using Cufflinks 2.1.1 [30]. Among the 14,592 annotated genes expressed in the two samples, 13,875 were previously annotated genes and 717 were novel genes (Additional file 4). According to the improved genome annotation, the transcriptional activity of the genes was estimated.

### Functional classification of *A. flavus* DEGs in response to resveratrol

The differently expressed genes between the two libraries provide a clue to the molecular mechanism related to the *A. flavus* response to resveratrol. By comparing expression levels in resveratrol-treated and untreated samples, we identified 453 genes that were significantly differentially transcribed (log_2_[fold change] = log_2_[AM-Res/AM] > 1, *q* value < 0.005) between the two samples, including 22 genes (4.86 % of the DEGs) showing a 10-fold change (Additional file 5). Of these, 366 genes (80.79 %) were up-regulated and 87 (19.21 %) genes were down-regulated. Genes with altered expression were related to a wide variety of regulatory and metabolic processes.

Functional assignments were defined by Gene Ontology (GO) terms (http://www.genontology.org/), which provided a broad functional classification of genes and gene products for various biological processes, molecular functions, and cellular localizations. GO functional enrichment analysis revealed that these significantly DEGs were mainly involved in fatty acid metabolic process, monocarboxylic acid metabolic process, signaling receptor activity, phosphorelay sensor kinase activity, protein histidine kinase activity, and phosphotransferase activity (nitrogenous group as acceptor) (*q* value < 0.05) (Table 2 and Additional file 6). As mentioned above, *A. flavus* and other *Aspergilli* preferentially colonize crop seeds with a high oil content because these crops contain high levels of unsaturated fatty acids, linoleic (18:2) and oleic acid (18:1), which are substrates for oxygenases [31, 32]. Oxylipins, derived from oxygenases, are a class of oxygenated, unsaturated fatty acids involved in signaling pathways in filamentous fungi, yeasts, oomycetes, plants, and animals [33]. A family of oxylipin-producing oxygenases and their products, which is encoded by *ppo* (fungi) and *lox* (plants, animals, and fungi), are involved in regulating sclerotia and conidia formation and secondary metabolism in *A. flavus*, *A. nidulans*, and *A. parasiticus* [5, 34]. Oxylipins could potentially interact with G-protein-coupled receptor complexes upstream of the heterotrimeric G-protein complex that regulates aflatoxin/sterigmatocystin production [35, 36]. Moreover, the transcription of genes of fatty acid synthases (AFLA_139380 and AFLA_139370), the key genes in the aflatoxin biosynthetic pathway cluster, was significantly down-regulated when *A. flavus* was treated with resveratrol.

**Table 2:** Gene Ontology (GO) functional enrichment analysis of differentially expressed genes when *A. flavus* was treated with resveratrol

GO ID	Term name	Name space	*p* value	*q* value	DEG	DEG
					list	item
GO:0006631	Fatty acid metabolic process	Biological process	1.19E-05	0.02473	11	257
GO:0032787	Monocarboxylic acid metabolic process	Biological process	2.52E-05	0.02473	12	257
GO:0038023	signaling receptor activity	Molecular function	2.77E-05	0.02473	7	257
GO:0000155	Phosphorelay sensor kinase activity	Molecular function	3.70E-05	0.02473	6	257
GO:0004673	Protein histidine kinase activity	Molecular function	3.70E-05	0.02473	6	257
GO:0016775	Phosphotransferase activity (nitrogenous group as acceptor)	Molecular function	3.70E-05	0.02473	6	257

*q* value: Corrected *p* value; DEG list: the number of GO-annotated differently expressed genes; DEG item: the number of differently expressed genes associated with the GO term

To further investigate the biological functions and interactions of genes, pathway-based analysis was conducted using the Kyoto Encyclopedia of Genes and Genomes (KEGG, http://www.genome.ad.jp/kegg) pathway database, which records the network of molecular interactions in the cells and variants specific to particular organisms. However, KEGG metabolic pathway analysis failed to confirm enrichment in any pathway in the RNA-seq data (*q* value < 0.05) obtained in this study, which is consistent with results of a previous study by Lin [10].

### Validation of RNA-seq analysis by quantitative real-time PCR (qRT-PCR)

**Fig. 5:**
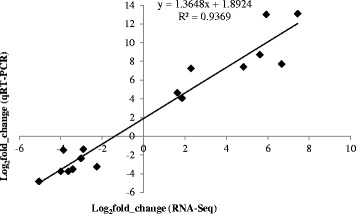
Quantitative real-time PCR validations of the up-regulated and down-regulated genes characterized by RNA-seq. log_2_(fold change) = log_2_(AM-Res/AM)

It is common practice to validate RNA-seq differential analysis with quantitative analysis of a subset of genes by qRT-PCR. Thus, we compared qRT-PCR expression levels of 16 DEGs randomly selected from the resveratrol-treated and untreated *A. flavus* samples (Table 3). Gene-specific primer pairs (Table 3) were designed according to the 16 genes’ sequences using the online Primer 3-BLAST program (bioinfo.ut.ee/primer3-0.4.0/primer3/, NCBI). The qRT-PCR data mirrored the quantification by RNA-seq (Fig. 5), with a significant correlation (R^2^ = 0.936), indicating the authenticity of these DEGs. Thus, these comparisons of data from qRT-PCR and RNA-seq of strain AF2202 not only validated the transcriptome analysis, but also reinforced the power of next-generation sequencing in quantifying the entire repertoire of genes.

**Table 3:** Primer pairs for each target gene for quantitative real-time PCR

Gene Name	Primer sequence (5′-3′)	Product size	Annealing temperature (°C)
		(bp)	
Up-regulated			
AFLA_125090	AGGTTGTTCTCGGTCTGGTT	137	65
	GCAAGGTCACCTACATGCAC		
AFLA_075280	AGCTGGTTCGGTTTACCATC	128	64.5
	ATGGCGATAGGGACAGGTAG		
AFLA_057960	TGCAGACCAATGTTCATCCT	108	64.5
	GTTGTCTCAGTCGTGCCAGT		
AFLA_039390	CGCAACAAAGCAAGACATTT	128	61.4
	GTCGGAGGGCTTGATTGTAT		
AFLA_077590	CGTCGATTATGATGGAGACG	65	58.9
	CACTGCTCAGCATTCCGTAT		
AFLA_021650	GTTGGGCTATACGGAGGTGT	137	58.9
	GCCATAGAGCAGCCAGTACA		
AFLA_075300	GTGATTCATAGGGCCGACTT	156	64.5
	GACGACAAGGTCTCCGGTAT		
AFLA_023780	CTCCTCGACCGATTACCATT	125	57.1
	CCCACTAACCACATCGACAG		
Down-regulated			
AFLA_117290	TTGCCCTCTGTTGAAGACTG	92	65
	CCTCGTAGGACTTCCTCAGC		
AFLA_112010	CAGTCGAACCCTATCCACCT	145	58.9
	TACGTTCGGAGACACGGATA		
AFLA_137320	AGCTACCCAGCACCAGAAGT	76	58.9
	TGTAGGCAGGAGTCTGTTCG		
AFLA_117420	CGGCAGAAGAGTACTGGTGA	106	57.1
	ACGACTGGTGGTGACGATAA		
AFLA_117340	TACAACCGGCTACAGCTCAC	113	57.1
	TGATCGACTCGGAAAGACTG		
AFLA_058990	ACGAGTCCTACCACCAGTCC	64	55
	TTGATGGTTCCTCCTCCTTC		
AFLA_128370	CCCTCTTTGGTAAGAATCGC	144	55.8
	CGTCGGCCATATTCACATAG		
AFLA_129450	GTACAGCCGTGGGATTCTTT	112	55.8
	TGGAGCCAGTAGATATTGCG

### Gene expression of the secondary metabolism gene clusters in response to resveratrol

The *A. flavus* genome sequence contains hallmark enzymatic genes associated with secondary metabolite synthesis, including 55 putative secondary metabolism gene clusters [24, 37, 38]. Each secondary metabolism gene cluster contains enzymatic genes and transcription factors for compound synthesis [39]. To evaluate the effect of resveratrol on the regulation of secondary metabolism, we used the information from the web-based software Secondary Metabolite Unknown Regions Finder (SMURF, http://www.jcvi.org/smurf) to identify the putative secondary metabolism gene clusters of *A. flavus*. Among the 55 clusters in the *A. flavus* genome, the expression levels of most clusters were not significantly affected by resveratrol (Additional file 7). By comparing the expression levels of secondary metabolic genes in resveratrol-treated and untreated samples, we identified 17 genes (2.45 % of 695 secondary metabolic genes) in 13 secondary metabolism gene clusters that were significantly differentially transcribed (log_2_[fold change] = log_2_[AM-Res/AM] > 1, *q* value < 0.005), with 2 backbone genes (AFLA_004450 and AFLA_118440) (Additional file 7). The backbone genes included a non-ribosomal peptide synthase (NRPS, AFLA_004450), which is an enzyme that produces a polypeptide independent of ribosomes, and an NRPS-like synthase (AFLA_118440), which was named because it does not contain all of the typical domains normally found in an NRPS [2].

In the aflatoxin biosynthetic pathway cluster, 30 genes were identified as cluster #54 [2]. In this study, the transcription of 23 of the 30 genes was depressed to various degrees with resveratrol treatment, and only 7 genes (AFLA_139460, AFLA_139450, AFLA_139390, AFLA_139260, AFLA_139330, AFLA_139220, and AFLA_139210) were not down-regulated (Additional file 7). Interestingly, *aflA* (AFLA_139380) and *aflB* (AFLA_139370) were significantly down-regulated (*q* value < 0.005). *aflA* and *aflB* encode the fatty acid synthetase alpha subunit (Fas-2) and beta subunit (Fas-1), respectively, which catalyze the reaction between acetyl-CoA and malonyl-CoA to form the starter unit hexanoate of aflatoxin biosynthesis [40, 41]. However, the transcription regulator genes *aflR* (AFLA_139360) and *aflS* (AFLA_139340) in the cluster showed only medium or slight down-regulation (Additional file 7), and the transcription of the global secondary metabolite regulators *laeA* (AFLA_033290) and *veA* (AFLA_066460) did not show significant depression (Table 4). Moreover, the expression of three genes (AFLA_139500, AFLA_139240, and AFLA_139230) was too low in the control sample to be distinguished by HTSeq (v0.5.3p9).

**Table 4:** Differentially expressed genes related to the development and conidial formation of *A. flavus*

Gene ID	Gene Name	RPKM		Log_2_(AM-Res/AM)	*q value*	DEGs	Annotated gene function
		AM	AM-Res				
CADAFLAG00011929	AFLA_101920	4.6010	1.5649	−1.5559	1.249E-88	Yes	Extracellular developmental signal biosynthesis protein FluG
CADAFLAG00007028	AFLA_131490	76.2979	53.7967	−0.5041			Hypothetical protein
CADAFLAG00007282	AFLA_134030	7.7607	3.5087	−1.1452			Developmental regulator FlbA development.
CADAFLAG00007610	AFLA_137320	64.9838	16.8385	−1.9483	2.173E-07	Yes	C2H2 conidiation transcription factor FlbC
CADAFLAG00003623	AFLA_017380	67.9510	41.5711	−0.7089			Hypothetical protein
CADAFLAG00011576	AFLA_098380	0.4394	0.4080	−0.1068			Conidial hydrophobin RodA/RolA
CADAFLAG00003311	AFLA_014260	0.0000	0.2077	_			Conidial hydrophobin RodB/HypB
CADAFLAG00003719	AFLA_018340	74.9734	43.1293	−0.7977			G-protein complex alpha subunit GpaA/FadA
CADAFLAG00006026	AFLA_046990	173.0674	78.9994	−1.1314	1.435E-55	Yes	APSES transcription factor StuA (stunted)
CADAFLAG00007519	AFLA_136410	142.1301	101.1580	−0.4906			Transcriptional regulator Medusa(medusa)
CADAFLAG00001065	AFLA_082850	0.9080	0.4216	−1.1068			C2H2 type conidiation transcription factor BrlA
CADAFLAG00002337	AFLA_029620	1.5396	0.2376	−2.6962			Transcription factor AbaA
CADAFLAG00011194	AFLA_052030	11.7189	5.8671	−0.9981			Regulatory protein wetA
CADAFLAG00006026	AFLA_046990	173.0674	78.9994	−1.1314			Cell pattern formation-associated protein stuA
CADAFLAG00002704	AFLA_033290	27.7370	19.7674	−0.4887	7.824E-10		Regulator of secondary metabolism LaeA
CADAFLAG00008070	AFLA_066460	16.3529	13.0959	−0.3204	1.54E-32		Developmental regulator AflYf / VeA
CADAFLAG00000929	AFLA_081490	33.9585	43.1005	0.3439			Nucleoside diphosphatase Gda1/VelB
CADAFLAG00002131	AFLA_027560	22.5485	37.5174	1.2854	1.77E-07	Yes	Vacuolar protein sorting-associated protein 13
CADAFLAG00003658	AFLA_017730	24.5015	41.4414	1.3091	0.0002471	Yes	Regulator of V-ATPase in vacuolar membrane protein 1
CADAFLAG00005274	AFLA_039470	27.8848	75.9818	1.9970	1.34E-11	Yes	Vacuolar calcium ion transporter
CADAFLAG00006698	AFLA_060890	137.9586	260.6940	1.4690	5.93E-15	Yes	Vacuolar amino acid transporter 3
CADAFLAG00010071	AFLA_130130	166.9408	312.7826	1.4567	6.59E-09	Yes	Putative vacuolar protein sorting-associated protein TDA6
Novel00107	Novel00107	32.5194	66.4116	1.5810	0.0032328	Yes	Vacuolar protein sorting-associated protein 13
CADAFLAG00011638	AFLA_099000	317.3651	906.9007	2.0657	1.19E-49	Yes	Superoxide dismutase [Cu-Zn]
CADAFLAG00010511	AFLA_004450	5.3195	1.3098	−1.4711	6.84E-09	Yes	Nonribosomal peptide synthetase 12

RPKM: Reads per kilobase per million reads; *q* value: corrected *p* value; DEGs: differently expressed genes; AM-Res (Treatment) and AM (Control): *A. flavus* was cultured in A&M medium with or without resveratrol, respectively; log_2_(fold_change) = log_2_(AM-Res/AM); log_2_(fold_change) > 0: the expression of a gene was up-regulated; log_2_(fold_change) < 0: the expression of a gene was down-regulated

In cluster #2, only the backbone gene AFLA_004450, encoding a dimethylallyl transferase (DMAT, an indole-alkaloids) enzyme, had significantly down-regulated transcription (*q* value < 0.005), but the transcription of the other 9 genes in this cluster was not significantly different when resveratrol was applied (Additional file 7). Also, in cluster #44, which has an unknown product, only the backbone gene AFLA_118440, encoding a polyketide synthetase (PKS) enzyme, was significantly up-regulated with resveratrol treatment (*q* value < 0.005) (Additional file 7). A total of 6 genes in cluster #9 (AFLA_017860 and AFLA_017820), #24 (AFLA_069370 and AFLA_069340), and #41 (AFLA_116250 and AFLA_116240) showed significant differential transcription (*q* value < 0.005) with resveratrol treatment. The products of these three clusters are unknown, but their backbone enzymes were predicted by SMURF. The backbone enzyme of cluster #9 is an NRPS-like enzyme, which is encoded by AFLA_017840. The backbone gene AFLA_069330 of cluster #24 encodes the NRPS Pes1, and the backbone enzyme of cluster #41 is a PKS. Genes in the other secondary metabolism gene clusters did not show significantly altered expression when resveratrol was applied. Only a few genes, such as AFLA_008690 in cluster #6, AFLA_054050 in cluster #16, AFLA_059950 in cluster #18, AFLA_060690 in cluster #19, AFLA_070820 in cluster #25, AFLA_084160 in cluster #30, and AFLA_108440 in cluster #37, were significantly affected (*q* value < 0.005) (Additional file 7).

### Expression analysis of *A. flavus* growth- and conidial development-related genes in response to resveratrol

Using the expression profiles of *A. flavus* in different treatments, especially the 453 genes that were significantly differentially transcribed (Additional file 5), we observed that some genes involved in mycelial and conidial development were down-regulated when resveratrol was applied (Table 4). Noticeably, the transcription of the so-called fluffy gene family (AFLA_101920, AFLA_137320, AFLA_134030, AFLA_131490, and AFLA_017380), whose null mutants have a fluffy or floccose phenotype [25], was down-regulated to various degrees (Table 4). *fluG* (AFLA_101920) and *flbC* (AFLA_137320) showed significantly depressed transcriptional levels (*q* value < 0.005) when *A. flavus* was treated with resveratrol. *fluG* encodes an extracellular development signal protein, which may function as a GSI-related enzyme in the synthesis of a small diffusible factor that acts as an extracellular signal directing asexual sporulation and other aspects of colony growth in *A. nidulans* [42]. *flbC* encodes a zinc finger protein (FlbC) that is required for morphogenesis and the elongation of flagellar filaments by facilitating polymerization of the flagellin monomers at the tip of a growing filament in *Escherichia coli* [43]. Concurrently, some conidia-specific genes (AFLA_098380 and AFLA_014260) were down-regulated slightly (Table 4). The molecular action of conidial hydrophobins RodA/RolA (AFLA_098380) and RodB/HypB (AFLA_014260) contributed to the structural integrity of the cell wall [44, 45]. In addition, the transcription of genes related to conidial development, such as *BrlA* (AFLA_082850), *AbaA* (AFLA_029620), *wetA* (AFLA_052030), *stuA* (AFLA_046990), *GpaA*/*FadA* (AFLA_018340), and *Medusa* (AFLA_136410), was decreased to a certain extent (Table 4). Interestingly, transcription of the *stuA* gene (AFLA_046990), which encodes the APSES transcription factor StuA that affects the orderly differentiation and spatial organization of cell types of the conidiospore, was significantly decreased (the RPKM value decreased from 173.07 to 79.00, *q* value < 0.005) [46].

We also found that the transcription of the gene AFLA_099000 was significantly increased. In *A. oryzae*, AFLA_099000 encodes a superoxide dismutase [Cu-Zn] that destroys radicals within the cells and is toxic to biological systems (Table 4). Interestingly, the transcription of genes involved in vacuolar proteins, such as AFLA_027560, AFLA_017730, AFLA_039470, AFLA_060890, AFLA_130130, and Novel 100107, was significantly increased (Table 4). The vacuolar proteins may take advantage of the exchange of atmosphere and nutrients between the organism and the environment. Nonribosomal peptide synthetase (NRPS) is a key factor responsible for the biosynthesis of bioactive metabolites that potentially contribute to organismal virulence; NRPS is encoded by AFLA_004450, which was significantly down-regulated (Table 4).

## Discussion

Considerable progress has been achieved in reducing aflatoxin contamination in agricultural products [47–52] since the discovery that aflatoxin is extremely toxic to humans and animals [2, 53]. Many inhibitors [9, 54] have been demonstrated to have an inhibitory effect on aflatoxin biosynthesis in *A. flavus*. Among them, plant-derived metabolites are of special interest. Non-host plant metabolites, such as onion, garlic extracts, eugenol [55–57], khellin, visnagin [58], caffeine, and piperlongumine [59] inhibit *A. flavus* growth and/or aflatoxin production. At the same time, host plant-derived metabolites including luteolin [60], eriodictyol [61], and tanning acids [62] also inhibit *A. flavus* development and/or aflatoxin production. However, most of these compounds can hardly be applied in practice because their biosynthesis pathway and the related biochemical steps are not well understood.

Resveratrol, a natural phytoalexin, could protect plants from fungal infection and improve the defense capacity in plants [63]. Some studies indicated that phytoalexins play an important role in resistance to aflatoxin contamination in peanut under drought stress [64], and the isoflavone phytoalexin glyceollin from soybean could inhibit aflatoxin production [65]. Field experiments demonstrated that a correlation existed between increased phytoalexins and decreased aflatoxin B_1_ [66]. The results from our previous experiments demonstrated that the resveratrol level was a factor that was relevant to aflatoxin resistance in peanut [12]. Resveratrol could affect *A. flavus* development and inhibit conidia formation and aflatoxin production. Moreover, the resveratrol biosynthetic pathway is well known [67]. Thus, it is possible to enhance resveratrol biosynthesis and concentration by breeding or genetic engineering, which may provide an applicable means for aflatoxin contamination management. Some transgenic studies indicated that resveratrol could inhibit other pathogenic fungi [68, 69]. In addition, resveratrol has been suggested to have a beneficial effect on human health [70, 71]. Genetic improvement for an increased resveratrol content in crops could not only ensure food safety, but also enhance the nutritional value of agricultural products.

Aflatoxins are polyketide-derived difurano cumarins, their biosynthesis involves at least 18 enzymatic reactions [72, 73]. The first step in the biosynthesis requires the formation of the starter unit hexanoate from acetyl-CoA and malonyl-CoA catalyzed by Fas-1 and Fas-2 [40, 41], which is then catalyzed by PksA, a polyketide synthase encoded by *aflC*, to produce norsolorinic acid anthrone (NAA). The monooxygenase HypC oxidizes the intermediate NAA to form anthraquinone norsolorinic acid, which is the first stable intermediate in aflatoxin biosynthesis [74, 75]. In this study, we found that *aflA* (*Fas*-*2*) and *aflB* (*Fas*-*1*) in the aflatoxin biosynthesis pathway cluster were significantly down-regulated when *A. flavus* was treated with resveratrol. Aflatoxin is a highly oxygenated product, and oxidative stress is a prerequisite for aflatoxin production [76]. Resveratrol-inhibited aflatoxin biosynthesis also has antioxidant activity. Superoxide dismutase [Cu-Zn] (AFLA_099000) (Table 4) had a significantly increased expression level when resveratrol was added to medium; it destroyed radicals that were normally produced within the cells and were toxic to biological systems. Superoxide dismutases inhibit free radical scavenging and protect calcineurin (a Ca^2+^/calmodulin-dependent protein phosphatase) from inactivation [77]. The activity of calcineurin was enhanced at periods corresponding to aflatoxin production, and aflatoxin itself can inhibit calmodulin kinase activity [78]. Calmodulin also activated acetyl-CoA carboxylase, a key enzyme in aflatoxin biosynthesis [79]. The transcription of genes involved in vacuolar proteins significantly increased (Table 4); these proteins may be involved in the exchange of atmosphere and nutrients between the organism and the environment. Moreover, 23 of the 30 genes in the aflatoxin metabolic cluster had depressed transcription levels. These observations suggested that resveratrol directly inhibits the expression of the aflatoxin pathway cluster genes *aflA* and *aflB*, which could significantly suppress the formation of the starter unit (hexanoate) of aflatoxin biosynthesis. In addition, resveratrol highly increased the activity of antioxidative enzymes to limit aflatoxin production. There was also a certain amount of aflatoxin produced by *A. flavus* treated with resveratrol because aflatoxin biosynthesis was only partially inhibited rather than completely suppressed.

Based on our experimental data, resveratrol could influence the mycelia development and conidia formation of *A. flavus* (Figs. 1 and 2, Table 1), as well as several other biochemical substances previously reported [10, 80, 81]. Additionally, we have found that some genes involved in mycelial and conidial development were down-regulated when *A. flavus* was treated with resveratrol (Table 4). The changes in mycelia (the morphological characteristics of mycelia in liquid medium and the myceloid colony on solid medium) was affected by resveratrol, which may be related to the low-level expression of the so-called fluffy gene family. The formation of conidia, the asexual reproductive structure, in *A. flavus* requires the concerted activity of a number of signaling proteins and transcription factors [25]. Transcription of the conidia-specific genes *RodA*/*RolA* and *RodB*/*HypB* was down-regulated slightly when resveratrol was applied (Table 4), but the transcription of some genes related to conidial development showed various levels of depression when *A. flavus* was treated with resveratrol (Table 4). BrlA, AbaA, WetA, and StuA are pivotal regulators of conidiophore development and conidia maturation; they act individually and together to regulate their own expression and that of numerous other sporulation-related genes. The transcriptomic profiling showed that resveratrol significantly depressed the transcriptional levels of *stuA*, *fluG*, *flbC*, and other genes related to conidial development. BrlA, a Cys_2_His_2_-type conidiation transcription factor, mediates the developmental switch from the apical growth pattern of vegetative cells to the budding growth of conidiophores; *brlA* mutants cannot produce conidia [25]. The transcription factor AbaA controls the temporal and spatial specificity of development in *Aspergillus*. The expression of *abaA* leads to the activation of *brlA* and *wetA* and to the cessation of growth [82]. The regulatory protein WetA is responsible for activating a set of genes whose products make up the final two conidial wall layers or direct their assembly [82]. The APSES transcription factor StuA (stunted) is required for the orderly differentiation and spatial organization of cell types of the conidiophores. StuA affected the spatial localization of AbaA and BrlA, and the expression of *StuA* is dependent on a functional BrlA protein [46]. The transcript of *GpaA*/*FadA* was involved in conidial development. A previous study demonstrated that *ΔgprA* and *ΔgprD* mutants did not exhibit hyper-conidiation when exposed to 0.5 and 1.0 mg fatty acid-soaked discs [83]. The transcriptomic profiling showed that resveratrol significantly depressed the transcriptional levels of *stuA*, *fluG*, *flbC*, and other genes related to conidial development. These results demonstrated that resveratrol greatly reduced conidia formation of *A. flavus* by affecting gene expression at the transcriptomic level.

Numerous studies indicated that conidial development and secondary metabolism are co-regulated [84–87]. The heterotrimeric nuclear complex (VelB/VeA/LaeA complex, Velvet complex) was demonstrated to be a global regulator of fungal development and secondary metabolism in *Aspergillus* [10, 84, 85, 88]. *laeA* and *veA* are two critical genes in the formation of the Velvet complex: VeA bridges VelB and LaeA to form the complex [2, 88]. In our data, the transcription of *laeA* and *veA* were slightly depressed in the *A. flavus* samples treated with resveratrol (Table 4), which is consistent with the results of previous studies. Microarray data provided evidence for LaeA regulation of 24 clusters, including the aflatoxin biosynthetic cluster, out of 55 secondary metabolism clusters in *A. flavus* [87]. VeA regulates aflatoxin, aflatrem, and cyclopiazonic acid production [1]. In addition, null mutants of *laeA* and *veA* produced fewer conidia and less aflatoxin in host seeds [2, 86]. This indicated that the inhibition of *laeA* and *veA* transcript levels by resveratrol may lead to a loss of toxin production and conidia formation.

## Conclusions

Resveratrol treatment of *A. flavus* decreased aflatoxin production and conidia formation, and it also caused abnormal mycelia development. First, resveratrol directly inhibited the expression of aflatoxin biosynthetic pathway cluster genes, the *aflA* and *aflB* expression at significantly low transcriptional levels could result in an insufficient amount of the starter unit hexanoate for aflatoxin biosynthesis. In addition, resveratrol could significantly enhance the activity of antioxidative enzymes, which destroy radicals produced within the cells that are toxic to biological systems, leading to decreased aflatoxin production. Second, the decreased transcription of *fluG* and *flbC* could affect the mycelia (colony) development and asexual sporulation. The altered appearance of mycelia and mycelioid colonies could be a result of the depressed transcription of the so-called fluffy gene family. Third, resveratrol effectively decreased conidia formation of *A. flavus*, which may result from the suppressed transcription of genes related to conidial development. The low level of transcription of *stuA*, *brlA*, *wetA*, and *abaA*, the pivotal regulators of conidial development and maturation, as well as other genes related to conidial development, disrupted the cell’s orderly differentiation and spatial organization of conidia, blocking conidia formation. Fourth, the inhibition of *laeA* and *veA* by resveratrol may lead to a loss of aflatoxin production and conidia formation, which further demonstrated that conidial development and secondary metabolism are co-regulated.

## Methods

### Effects of resveratrol treatment on *A. flavus*

A toxigenic *A. flavus* strain (AF2202) isolated from peanut was maintained at Oil Crops Research Institute of Chinese Academy of Agricultural Sciences in glycerol 20 % at −80 °C. To prepare the *A. flavus* inoculum, some conidia of AF2202 were taken from the stored sample and cultured on fresh potato dextrose agar medium at 29 ± 1 °C for 7 days. Conidia were then collected and suspended in sterile water containing 0.05 % Tween-80. The concentration of conidia in the suspension was determined using a haemocytometer.

Liquid A&M medium with a final resveratrol (Sigma®) concentration of 3.0 μg/mL [12] was prepared and added to conical 100-mL flasks containing 60 mL medium. Liquid A&M medium without resveratrol was used as a control. Each flask was inoculated with 5 × 10^5^ colony-forming units (CFU) *A. flavus* conidia (freshly prepared). The inoculated flasks were incubated at 30 °C with continuous shaking (160 rpm) for 3 or 7 days. After 3 days of incubation, the fungal mycelia were collected by filtration and stored at −80 °C for RNA extraction. After 7 days of incubation, fungal mycelia were also collected by filtration, and the biomass was measured after drying overnight at 60 °C. The dried mycelia were then ground to fine powder and put back into the corresponding flasks. Then, 25 mL chloroform was added to each flask and incubated with continuous shaking (200 rpm) for 30 min at room temperature. Five milliliters of the chloroform layer was added to a test tube and dried under nitrogen gas. The residue was dissolved in 1.0 mL methanol. Then, aflatoxins B_1_ and B_2_ were tested using high-performance liquid chromatography as previously described [12].

Solid A&M medium was prepared by adding agar to the above liquid medium with or without resveratrol (1.5 g agar was added to 100 mL liquid medium). The solid medium was used to investigate the effect of resveratrol on conidia formation and mycelia growth of *A. flavus*. A suspension with 1.6 × 10^5^ conidia CFU was inoculated on a 90-mm diameter Petri dish containing 20 mL solid medium. The inoculated Petri dishes were incubated at 30 °C in darkness for 7 days. For each dish, the conidia were suspended, the concentration was determined using a haemocytometer, and the colony diameter was measured.

Three replications were performed for each treatment in the above experiments.

### RNA isolation and cDNA library construction

Aflatoxin was initially detected at 2 days of *A. flavus* (AF2202) growth in liquid A&M medium. The aflatoxin level increased at maximum rates between 3 and 4 days and then increased slowly after that point. However, the aflatoxin production rate in the resveratrol-treated culture (3.0 μg/mL) was much lower than that in the control when *A. flavus* was cultured between 3 and 4 days (Additional file 8). Additionally, the conidia were initially tested at 3 days of AF2202 growth on solid A&M medium. The morphological changes of *A. flavus* suggested that resveratrol could affect the expression of genes that are related to aflatoxin production and conidia formation. Therefore, we sampled the mycelium to isolate RNA and constructed cDNA libraries when AF2202 was cultured for 3 days.

Total RNA of AF2202 was isolated using the RNeasy® Plant Mini Kit (QIAGEN) according to the manufacturer’s protocol. Each pooled RNA sample for cDNA library construction was composed of three individual RNA samples with equal weight extracted from mycelia that were independently cultured. All RNA samples were treated with RNase-free DNase I. The concentration and integrity of the pooled total RNA was checked using a NanoDrop® 1000 spectrophotometer, a Qubit® Fluorometer 2.0, and an Agilent 2100 bioanalyzer to confirm that all samples had an RNA integrity number value above 9.0.

Sequencing libraries were constructed using the Illumina TruSeq™ RNA Sample Preparation Kit (Illumina) following the manufacturer’s instructions. Four index codes were added to attribute sequences to each sample. The mRNA was isolated from total RNA using poly-T oligo-attached magnetic beads. Fragmentation was carried out using divalent cations under elevated temperature in Illumina proprietary fragmentation buffer. First-strand cDNA was synthesized using random oligonucleotides and SuperScript II. Second-strand cDNA synthesis was subsequently performed using DNA polymerase I and RNase H. Remaining overhangs were converted into blunt ends via exonuclease/polymerase, and then enzymes were inactivated. After adenylation of 3′ ends of cDNA fragments, Illumina PE adapter oligonucleotides were ligated for hybridization. The cDNA fragments of 200 bp in length were isolated with the AMPure XP system (Beckman Coulter). DNA fragments with ligated adapter molecules on both ends were selectively enriched using Illumina PCR Primer Cocktail in a 15-cycle PCR. Products were purified with the AMPure XP system and quantified using the Agilient high-sensitivity DNA assay with the Agilent Bioanalyzer 2100 system.

Clustering of the index-coded samples was performed with a cBot Cluster Generation System using a TruSeq PE Cluster Kit (v3-cBot-HS, Illumina) according to the manufacturer’s instructions. After cluster generation, the prepared libraries were sequenced with the Illumina Hiseq 2000 platform, and then 100-bp paired-end reads were generated.

### Mapping reads to the reference genome and quantifying gene expression

Raw data (raw reads) of fastq format were first processed using in-house perl scripts. Clean data (clean reads) were obtained by removing reads containing adapter, reads containing poly-N, and low-quality reads from the raw data. The Q20, Q30, GC content, and sequence duplication level of the clean data were calculated (Additional 1 and Fig. 3). All downstream analysis used the clean data with high quality.

The *A. flavus* (NRRL 3357) genome and gene model annotation files were downloaded from the genome website (ftp://ftp.ensemblgenomes.org/pub/release-17/fungi/fasta/Aspergillus_flavus/dna/) directly. The index of the reference genome was built using Bowtie (v2.0.6) [89], and paired-end clean reads were aligned to the reference genome using Tophat (v2.0.7) with “mismatch 2” as the parameter [90]. HTSeq (v0.5.3p9) (http://www-huber.embl.de/users/anders/HTSeq) was used to count the read numbers mapped to each gene. Then, the RPKM of each gene was calculated based on the length of the gene and read count mapped to this gene [29].

### Identification and analysis of DEGs

Prior to differential gene expression analysis, the read counts for each sequenced library were adjusted using the edgeR program package (TMM) with one scaling normalization factor. Differential expression analysis of two conditions (with and without resveratrol treatment) was performed using DEGSeq (1.2.2) [91] and edgeR (3.2.4) [92]. The *p* values were adjusted with the Benjamini and Hochberg method [93], and a corrected *p* value (*q* value) of 0.005 and log_2_ (fold change) of 1 were set as the threshold for significantly differential expression [94].

GO (http://www.geneontology.org/) is an international standardized gene function classification system that offers a dynamically updated, controlled vocabulary and strictly defined concepts to comprehensively describe properties of genes and their products in any organism. In our experiments, GO enrichment analysis of DEGs was implemented using GOseq (Release 2.12) [95], in which the gene length bias was corrected. GO terms with a corrected *p* value (*q* value) less than 0.05 were considered to be significantly enriched by DEGs.

KEGG is the major public pathway-related database [96] used to identify significantly enriched metabolic pathways or signal transduction pathways in DEGs compared to whole-genome background (http://www.genome.jp/kegg/). We used KOBAS software (v 2.0) [97] to test the statistical enrichment of DEGs in KEGG pathways. KEGG terms with a corrected *p* value (*q* value) less than 0.05 were considered to be significantly enriched in DEGs.

### qRT-PCR analysis

To validate the repeatability and reproducibility of gene expression data obtained by RNA-seq in *A. flavus*, we randomly selected 16 DEGs for validation by qRT-PCR, as described previously [13, 25, 98, 99]. Gene-specific primer pairs (Table 2) were designed according to the sequences of the 16 genes using the Primer 3-BLAST program available online (bioinfo.ut.ee/primer3-0.4.0/primer3/, NCBI). To ensure accuracy, each primer was run with three replications on the same plate with a negative control that lacked template cDNA to detect non-specific products. For normalization, the *A. flavus* 18S rRNA gene was selected as the endogenous reference gene. The relative expression levels of genes were calculated using the 2^-ΔΔCt^ method [100, 101], which represents the C_T_ difference between the reference 18S rRNA and the target gene products. Three replications were performed for each qRT-PCR.

### Availability of supporting data

The sequencing data generated in this study have been deposited in NCBI’s Short Read Archive database (SRA, http://www.ncbi.nlm.nih.gov/Traces/sra_sub/sub.cgi) and are accessible through SRA series accession number SRP057550 (BioProject ID: PRJNA281697).

## Additional files

Additional file 1:
**Summary of RNA-sequencing reads in the study.** (DOCX 20 kb) Additional file 2:
**Summary of gene expression levels in two** ***Aspergillus flavus*** **samples.** (DOCX 16 kb) Additional file 3:
**Gene expression of the** ***A. flavus*** **genome. **RPKM: reads per kilobase per million reads; *q* value: corrected *p* value; log_2_(fold_change) = log_2_(AM-Res/AM). (XLSX 1280 kb) Additional file 4:
**Expression of novel genes.** RPKM: reads per kilobase per million reads; *q* value: corrected *p* value; log_2_(fold_change) = log_2_(AM-Res/AM). (XLSX 197 kb) Additional file 5:
**Differentially expressed genes of** ***A. flavus*** **in response to resveratrol.** log_2_(fold_change) = log_2_(AM-Res/AM); *q* value: corrected *p* value. (XLS 118 kb) Additional file 6:
**Histogram presentation of Gene Ontology (GO) enrichment analysis of differentially expressed genes.** The x-axis indicates the number of genes in a GO term; * indicates the significantly differentially expressed GO term. (DOC 367 kb) Additional file 7:
**Gene expression and differentially expressed genes in secondary metabolism gene clusters of** ***A. flavus***. RPKM: reads per kilobase per million reads; *q* value: corrected *p* value; log_2_(fold_change) = log_2_(AM-Res/AM). (XLS 175 kb) Additional file 8:
**The dynamic change of aflatoxin content in the mediun with and without resveratrol.** AM-Res (Treatment): *A. flavus* was cultured in A&M medium with resveratrol; AM (Control): *A. flavus* was cultured in A&M medium without resveratrol. (DOCX 14 kb)

## References

[1] Duran RM, Cary JW, Calvo AM (2007). Production of cyclopiazonic acid, aflatrem, and aflatoxin by *Aspergillus flavus* is regulated by veA, a gene necessary for sclerotial formation. Appl. Microbiol. Biotechnol..

[2] Amaike S, Keller NP (2011). Aspergillus flavus. Annu. Rev. Phytopathol..

[3] Klich MA (2007). *Aspergillus flavus*: the major producer of aflatoxin. Mol. Plant Pathol..

[4] Williams JH, Phillips TD, Jolly PE, Stiles JK, Jolly CM, Aggarwal D (2004). Human aflatoxicosis in developing countries: a review of toxicology, exposure, potential health consequences, and interventions. Am J Clin Nutrit.

[5] Burow GB, Nesbitt TC, Dunlap J, Keller NP (1997). Seed lipoxygenase products modulate *Aspergillus* mycotoxin biosynthesis. Mol. Plant Microbe Interact..

[6] Goodrichtanrikulu M, Mahoney NE, Rodriguez SB (1995). The plant growth regulator methyl jasmonate inhibits aflatoxin production by *Aspergillus flavus*. Microbiol Uk.

[7] Chen ZY, Brown RL, Damann KE, Cleveland TE (2007). Identification of maize kernel endosperm proteins associated with resistance to aflatoxin contamination by *Aspergillus flavus*. Phytopathology.

[8] Wang T, Zhang E, Chen X, Li L, Liang X (2010). Identification of seed proteins associated with resistance to pre-harvested aflatoxin contamination in peanut (*Arachis hypogaea* L). BMC Plant Biol..

[9] Holmes RA, Boston RS, Payne GA (2008). Diverse inhibitors of aflatoxin biosynthesis. Appl. Microbiol. Biotechnol..

[10] Lin J-Q, Zhao X-X, Zhi Q-Q, Zhao M, He Z-M (2013). Transcriptomic profiling of *Aspergillus flavus* in response to 5-azacytidine. Fungal Genet. Biol..

[11] Chung IM, Park MR, Chun JC, Yun SJ (2003). Resveratrol accumulation and resveratrol synthase gene expression in response to abiotic stresses and hormones in peanut plants. Plant Sci..

[12] Wang H, Huang J, Lei Y, Yan L, Wang S, Jiang H, Ren X, Lou Q, Liao B (2012). Relationship of resveratrol content and resistance to aflatoxin accumulation caused by *Aspergillus flavus* in peanut seeds. Acta Agronomica Sinica.

[13] Wang B, Guo G, Wang C, Lin Y, Wang X, Zhao M, Guo Y, He M, Zhang Y, Pan L (2010). Survey of the transcriptome of *Aspergillus oryzae* via massively parallel mRNA sequencing. Nucleic Acids Res..

[14] Nagalakshmi U, Wang Z, Waern K, Shou C, Raha D, Gerstein M, Snyder M (2008). The transcriptional landscape of the yeast genome defined by RNA sequencing. Science.

[15] Wilhelm BT, Marguerat S, Watt S, Schubert F, Wood V, Goodhead I, Penkett CJ, Rogers J, Bahler J (2008). Dynamic repertoire of a eukaryotic transcriptome surveyed at single-nucleotide resolution. Nature.

[16] Linz JE, Wee J, Roze LV (2014). *Aspergillus parasiticus* SU-1 Genome Sequence, Predicted Chromosome Structure, and Comparative Gene Expression under Aflatoxin-Inducing Conditions: Evidence that Differential Expression Contributes to Species Phenotype. Eukaryot. Cell.

[17] Chang PK, Scharfenstein LL, Mack B, Yu JJ, Ehrlich KC (2014). Transcriptomic profiles of *Aspergillus flavus* CA42, a strain that produces small sclerotia, by decanal treatment and after recovery. Fungal Genet. Biol..

[18] McGettigan PA (2013). Transcriptomics in the RNA-seq era. Curr. Opin. Chem. Biol..

[19] Maher CA, Kumar-Sinha C, Cao X, Kalyana-Sundaram S, Han B, Jing X, Sam L, Barrette T, Palanisamy N, Chinnaiyan AM (2009). Transcriptome sequencing to detect gene fusions in cancer. Nature.

[20] Martin JA, Wang Z (2011). Next-generation transcriptome assembly. Nat. Rev. Genet..

[21] Payne GA, Nierman WC, Wortman JR, Pritchard BL, Brown D, Dean RA, Bhatnagar D, Cleveland TE, Machida M, Yu J (2006). Whole genome comparison of *Aspergillus flavus* and A-oryzae. Med. Mycol..

[22] Chang P-K, Ehrlich KC (2010). What does genetic diversity of *Aspergillus flavus* tell us about *Aspergillus oryzae*?. Int. J. Food Microbiol..

[23] Keller NP, Turner G, Bennett JW (2005). Fungal secondary metabolism - From biochemistry to genomics. Nat. Rev. Microbiol..

[24] Khaldi N, Seifuddin FT, Turner G, Haft D, Nierman WC, Wolfe KH, Fedorova ND (2010). SMURF: Genomic mapping of fungal secondary metabolite clusters. Fungal Genet. Biol..

[25] Wilkinson JR, Kale SP, Bhatnagar D, Yu J, Ehrlich KC (2011). Expression Profiling of Non-Aflatoxigenic *Aspergillus parasiticus* Mutants Obtained by 5-Azacytosine Treatment or Serial Mycelial Transfer. Toxins.

[26] Zhang F, Guo Z, Zhong H, Wang S, Yang W, Liu Y, Wang S (2014). RNA-Seq-Based Transcriptome Analysis of Aflatoxigenic *Aspergillus flavus* in Response to Water Activity. Toxins.

[27] Yu J, Fedorova ND, Montalbano BG, Bhatnagar D, Cleveland TE, Bennett JW, Nierman WC (2011). Tight control of mycotoxin biosynthesis gene expression in *Aspergillus flavus* by temperature as revealed by RNA-Seq. Fems Microbiol Lett.

[28] Hegedus Z, Zakrzewska A, Agoston VC, Ordas A, Racz P, Mink M, Spaink HP, Meijer AH (2009). Deep sequencing of the zebrafish transcriptome response to mycobacterium infection. Mol. Immunol..

[29] Mortazavi A, Williams BA, McCue K, Schaeffer L, Wold B (2008). Mapping and quantifying mammalian transcriptomes by RNA-Seq. Nat. Methods.

[30] Trapnell C, Williams BA, Pertea G, Mortazavi A, Kwan G, van Baren MJ, Salzberg SL, Wold BJ, Pachter L (2010). Transcript assembly and quantification by RNA-Seq reveals unannotated transcripts and isoform switching during cell differentiation. Nat. Biotechnol..

[31] Calvo AM, Hinze LL, Gardner HW, Keller NP (1999). Sporogenic effect of polyunsaturated fatty acids on development of *Aspergillus* spp. Appl. Environ. Microbiol..

[32] Feussner I, Wasternack C (2002). The lipoxygenase pathway. Annu. Rev. Plant Biol..

[33] Funk CD (2001). Prostaglandins and leukotrienes: Advances in eicosanoid biology. Science.

[34] Brown SH, Zarnowski R, Sharpee WC, Keller NP (2008). Morphological transitions governed by density dependence and lipoxygenase activity in *Aspergillus flavus*. Appl. Environ. Microbiol..

[35] Hicks JK, Yu JH, Keller NP, Adams TH (1997). *Aspergillus* sporulation and mycotoxin production both require inactivation of the FadA G alpha protein-dependent signaling pathway. Embo J.

[36] Brodhagen M, Keller NP (2006). Signalling pathways connecting mycotoxin production and sporulation. Mol. Plant Pathol..

[37] Cleveland TE, Yu J, Fedorova N, Bhatnagar D, Payne GA, Nierman WC, Bennett JW (2009). Potential of *Aspergillus flavus* genomics for applications in biotechnology. Trends Biotechnol..

[38] Georgianna DR, Fedorova ND, Burroughs JL, Dolezal AL, Bok JW, Horowitz-Brown S, Woloshuk CP, Yu J, Keller NP, Payne GA (2010). Beyond aflatoxin: four distinct expression patterns and functional roles associated with *Aspergillus flavus* secondary metabolism gene clusters. Mol. Plant Pathol..

[39] Hoffmeister D, Keller NP (2007). Natural products of filamentous fungi: enzymes, genes, and their regulation. Nat. Prod. Rep..

[40] Mahanti N, Bhatnagar D, Cary JW, Joubran J, Linz JE (1996). Structure and function of fas-1A, a gene encoding a putative fatty acid synthetase directly involved in aflatoxin biosynthesis in *Aspergillus parasiticus*. Appl. Environ. Microbiol..

[41] Minto RE, Townsend CA (1997). Enzymology and molecular biology of aflatoxin biosynthesis. Chem. Rev..

[42] Lee BN, Adams TH (1994). The Aspergillus nidulans fluG gene is required for production of an extracellular developmental signal and is related to prokaryotic glutamine synthetase I. Genes Dev.

[43] Kuwajima G, Asaka J, Fujiwara T, Node K, Kondo E (1986). Nucleotide sequence of the hag gene encoding flagellin of *Escherichia coli*. J. Bacteriol..

[44] Stringer MA, Dean RA, Sewall TC, Timberlake WE (1991). Rodletless, a new *Aspergillus* developmental mutant induced by directed gene inactivation. Genes Dev.

[45] Fedorova ND, Khaldi N, Joardar VS, Maiti R, Amedeo P, Anderson MJ, Crabtree J, Silva JC, Badger JH, Albarraq A (2008). Genomic islands in the pathogenic filamentous fungus *Aspergillus fumigatus*. Plos Genetics.

[46] Miller KY, Wu J, Miller BL (1992). StuA is required for cell pattern formation in *Aspergillus*. Genes Dev.

[47] Weaver MA, Abbas HK, Falconer LL, Allen TW, Pringle HC, Sciumbato GL (2015). Biological control of aflatoxin is effective and economical in Mississippi field trials. Crop Prot..

[48] Waliyar F, Osiru M, Ntare BR, Kumar KVK, Sudini H, Traore A, Diarra B (2015). Post-harvest management of aflatoxin contamination in groundnut. World Mycotoxin J.

[49] Mauro A, Battilani P, Cotty PJ (2015). Atoxigenic *Aspergillus flavus* endemic to Italy for biocontrol of aflatoxins in maize. Biocontrol.

[50] Ehrlich KC, Moore GG, Mellon JE, Bhatnagar D (2015). Challenges facing the biological control strategy for eliminating aflatoxin contamination. World Mycotoxin J.

[51] Chen ZY, Rajasekaran K, Brown RL, Sayler RJ, Bhatnagar D (2015). Discovery and confirmation of genes/proteins associated with maize aflatoxin resistance. World Mycotoxin J.

[52] Torres AM, Barros GG, Palacios SA, Chulze SN, Battilani P (2014). Review on pre- and post-harvest management of peanuts to minimize aflatoxin contamination. Food Res. Int..

[53] Forgacs J, Carll WT (1962). Mycotoxicoses. Adv. Vet. Sci..

[54] Ma JY, Mo HZ, Chen Y, Ding D, Hu LB (2014). Inhibition of Aflatoxin Synthesis in *Aspergillus flavus* by Three Structurally Modified Lentinans. Int. J. Mol. Sci..

[55] Bilgrami KS, Sinha KK, Sinha AK (1992). Inhibition of aflatoxin production and growth of *Aspergillus flavus* bt eugenol and garlic extracts. Indian J Med Res Section B.

[56] Ehrlich K, Ciegler A (1985). Effect of phytate on aflatoxin formation by *Aspergillus parasiticus* grown on different grains. Mycopathologia.

[57] Mahoney N, Molyneux RJ (2004). Phytochemical inhibition of aflatoxigenicity in *Aspergillus flavus* by constituents of walnut (*Juglans regia*). J. Agric. Food Chem..

[58] Mabrouk SS, El-Shayeb NMA (1992). Inhibition of aflatoxin production in *Aspergillus flavus* natural coumarins chromones. World J. Microbiol. Biotechnol..

[59] Lee SE, Mahoney NE, Campbell BC (2002). Inhibition of aflatoxin B-1 biosynthesis by piperlongumine isolated from *Piper longum* L. J. Microbiol. Biotechnol..

[60] Norton RA (1999). Inhibition of aflatoxin B-1 biosynthesis in *Aspergillus flavus* by anthocyanidins and related flavonoids. J. Agric. Food Chem..

[61] DeLucca AJ, Palmgren MS, Daigle DJ (1987). Depression of aflatoxin production by flavonoid-type compounds from peanut shells. Phytopathology.

[62] Azaizeh HA, Pettit RE, Sarr BA, Phillips TD (1990). Effect of peanut tannin extracts on growth of *Aspergillus parasiticus* and aflatoxin production. Mycopathologia.

[63] Hain R, Bieseler B, Kindl H, Schroeder G, Stoecker R (1990). Expression of a stilbene synthase gene in nicotiana-tabacum results in synthesis of the phytoalexin resveratrol. Plant Mol. Biol..

[64] Dorner JW, Cole RJ, Sanders TH, Blankenship PD (1989). Interrelationship of kernel water activity, soil temperature, maturity, and phytoalexin production in preharvest aflatoxin contamination of drought-stressed peanuts. Mycopathologia.

[65] Song DK, Karr AL (1993). Soybean phytoalexin, glyceollin, prevents accumulation of aflatoxin B-1 in cultures of *Aspergillus flavus*. J. Chem. Ecol..

[66] Zeringue HJ (2002). Effects of methyl jasmonate on phytoalexin production and aflatoxin control in the developing cotton boll. Biochem. Syst. Ecol..

[67] Halls C, Yu O (2008). Potential for metabolic engineering of resveratrol biosynthesis. Trends Biotechnol..

[68] Fettig S, Hess D (1999). Expression of a chimeric stilbene synthase gene in transgenic wheat lines. Transgenic Res..

[69] Thomzik JE, Stenzel K, Stocker R, Schreier PH, Hain R, Stahl DJ (1997). Synthesis of a grapevine phytoalexin in transgenic tomatoes (*Lycopersicon esculentum* Mill.) conditions resistance against Phytophthora infestans. Physiol Mol Plant Pathol.

[70] Athar M, Back JH, Kopelovich L, Bickers DR, Kim AL (2009). Multiple molecular targets of resveratrol: Anti-carcinogenic mechanisms. Arch. Biochem. Biophys..

[71] Tili E, Michaille J-J (2011). Resveratrol, microRNAs, inflammation, and cancer. J Nucleic Acids.

[72] Yabe K, Nakajima H (2004). Enzyme reactions and genes in aflatoxin biosynthesis. Appl. Microbiol. Biotechnol..

[73] Abrar M, Anjum FM, Butt MS, Pasha I, Randhawa MA, Saeed F, Wagas K (2013). Aflatoxins: biosynthesis, occurrence, toxicity, and remedies. Crit. Rev. Food Sci. Nutr..

[74] Ehrlich KC (2009). Predicted Roles of the Uncharacterized Clustered Genes in Aflatoxin Biosynthesis. Toxins.

[75] Ehrlich KC, Li P, Scharfenstein L, Chang P-K (2010). HypC, the Anthrone Oxidase Involved in Aflatoxin Biosynthesis. Appl. Environ. Microbiol..

[76] Jayashree T, Subramanyam C (2000). Oxidative stress as a prerequisite for aflatoxin production by Aspergillus parasiticus. Free Radical Biol Med.

[77] Wang XT, Culotta VC, Klee CB (1996). Superoxide dismutase protects calcineurin from inactivation. Nature.

[78] Jayashree T, Rao JP, Subramanyam C (2000). Regulation of aflatoxin production by Ca2+/calmodulin-dependent protein phosphorylation and dephosphorylation. Fems Microbiol Lett.

[79] Rao JP, Subramanyam C (2000). Calmodulin mediated activation of acetyl-CoA carboxylase during aflatoxin production by *Aspergillus parasiticus*. Lett. Appl. Microbiol..

[80] Chalfoun SM, Pereira MC, Resende MLV, Angelico CL, da Silva RA (2004). Effect of powdered spice treatments on mycelial growth, sporulation and production of aflatoxins by toxigenic fungi. Ciencia E Agrotecnologia.

[81] Greene-McDowelle DM, Ingber B, Wright MS, Zeringue HJ, Bhatnagar D, Cleveland TE (1999). The effects of selected cotton-leaf volatiles on growth, development and aflatoxin production of *Aspergillus parasiticus*. Toxicon.

[82] Mirabito PM, Adams TH, Timberlake WE (1989). Interactions of three sequentially expressed genes control temporal and spatial specificity in *Aspergillus* development. Cell.

[83] Affeldt KJ, Brodhagen M, Keller NP (2012). *Aspergillus* Oxylipin Signaling and Quorum Sensing Pathways Depend on G Protein-Coupled Receptors. Toxins.

[84] Bok JW, Keller NP (2004). LaeA, a regulator of secondary metabolism in *Aspergillus* spp. Eukaryot. Cell.

[85] Bok JW, Noordermeer D, Kale SP, Keller NP (2006). Secondary metabolic gene cluster silencing in *Aspergillus nidulans*. Mol. Microbiol..

[86] Duran RM, Cary JW, Calvo AM (2009). The role of veA in *Aspergillus flavus* infection of peanut, corn and cotton. Open Mycol J.

[87] Kale SP, Milde L, Trapp MK, Frisvad JC, Keller NP, Bok JW (2008). Requirement of LaeA for secondary metabolism and sclerotial production in *Aspergillus flavus*. Fungal Genet. Biol..

[88] Bayram O, Krappmann S, Ni M, Bok JW, Helmstaedt K, Valerius O, Braus-Stromeyer S, Kwon N-J, Keller NP, Yu J-H (2008). VelB/VeA/LaeA complex coordinates light signal with fungal development and secondary metabolism. Science.

[89] Langmead B, Salzberg SL (2012). Fast gapped-read alignment with Bowtie 2. Nat. Methods.

[90] Trapnell C, Pachter L, Salzberg SL (2009). TopHat: discovering splice junctions with RNA-Seq. Bioinformatics.

[91] Wang L, Feng Z, Wang X, Wang X, Zhang X (2010). DEGseq: an R package for identifying differentially expressed genes from RNA-seq data. Bioinformatics.

[92] Robinson MD, McCarthy DJ, Smyth GK (2010). EdgeR: a Bioconductor package for differential expression analysis of digital gene expression data. Bioinformatics.

[93] Benjamini Y, Hochberg Y (1995). Controlling the false discovery rate - a practical and powerful approach to multiple testing. J Royal Stat Soc Series B-Methodological.

[94] Fang S-M, Hu B-L, Zhou Q-Z, Yu Q-Y, Zhang Z (2015). Comparative analysis of the silk gland transcriptomes between the domestic and wild silkworms. BMC Genomics.

[95] Young MD, Wakefield MJ, Smyth GK, Oshlack A (2010). Gene ontology analysis for RNA-seq: accounting for selection bias. Genome Biol..

[96] Saldanha AJ (2004). Java Treeview-extensible visualization of microarray data. Bioinformatics.

[97] Mao XZ, Cai T, Olyarchuk JG, Wei LP (2005). Automated genome annotation and pathway identification using the KEGG Orthology (KO) as a controlled vocabulary. Bioinformatics.

[98] Reverberi M, Punelli M, Scala V, Scarpari M, Uva P, Mentzen WI, Dolezal AL, Woloshuk C, Pinzari F, Fabbri AA (2013). Genotypic and Phenotypic Versatility of *Aspergillus flavus* during Maize Exploitation. Plos One.

[99] Song Z, Yin Y, Jiang S, Liu J, Chen H, Wang Z (2013). Comparative transcriptome analysis of microsclerotia development in *Nomuraea rileyi*. BMC Genomics.

[100] Livak KJ, Schmittgen TD (2001). Analysis of relative gene expression data using real-time quantitative PCR and the 2(T)(−Delta Delta C) method. Methods.

[101] Pfaffl MW (2001). A new mathematical model for relative quantification in real-time RT-PCR. Nucleic Acids Res..

