# Supplementary material for: Deep sequencing analysis of transcriptomes in Aspergillus flavus in response to resveratrol
Source: BMC Microbiol. 2015 Sep 16;15:182. doi: 10.1186/s12866-015-0513-6 (PMC4589122; doi:10.1186/s12866-015-0513-6)
Supplement: Additional file 1: — Summary of RNA-sequencing reads in the study. (DOCX 20 kb) [file 12866_2015_513_MOESM1_ESM.docx]

**Additional file 1** - **Summary of RNA-Seq reads in the study**

|  | **Count (million)** | | |  | **Proportion (%)** | | |
| --- | --- | --- | --- | --- | --- | --- | --- |
|  | **AM (CK)** | **AM-Res (Treatment)** |  |  | **AM (CK)** | **AM-Res (Treatment)** |  |
| Total clean reads | 50561156 | 51441686 |  |  | \ | \ |  |
| Mapped reads | 45577031 | 46843066 |  |  | 90.14% | 89.88% |  |
| Uniquely mapped reads | 45342143 | 45999155 |  |  | 89.68% | 89.42% |  |
| Multi-mapped reads | 234888 | 236632 |  |  | 0.46% | 0.46% |  |
|  |  |  |  |  |  |  |  |
| Total nucleotides | 5.06 G | 5.14 G |  |  | \ | \ |  |
| Uniquely mapped nucleotides | 4.53 G | 4.60 G |  |  | 89.5% | 89.4% |  |
|  |  |  |  |  |  |  |  |
| Reads uniquely mapped in exon junctions | 8040544 | 7659667 |  |  | 15.9% | 14.89% |  |
| Reads mapped in genes of NCBI database | 38757260 | 40078217 |  |  | 76.65% | 77.91% |  |
| Reads mapped in rRNA | 0 | 0 |  |  | 0 | 0 |  |
|  |  |  |  |  |  |  |  |
| Error rate | \ | \ |  |  | 0.035 | 0.035 |  |
| Q20 | \ | \ |  |  | 97.37 | 97.47 |  |
| Q30 | \ | \ |  |  | 91.46 | 91.69 |  |
| GC content | \ | \ |  |  | 52.33 | 52.39 |  |
