# Supplementary material for: Deep sequencing analysis of transcriptomes in Aspergillus flavus in response to resveratrol
Source: BMC Microbiol. 2015 Sep 16;15:182. doi: 10.1186/s12866-015-0513-6 (PMC4589122; doi:10.1186/s12866-015-0513-6)
Supplement: Additional file 2: — Summary of gene expression levels in two Aspergillus flavus samples. (DOCX 16 kb) [file 12866_2015_513_MOESM2_ESM.docx]

**Additional file 2** - **Summary of expressed gene amounts in two *A. flavus* samples**

| **RPKM Interval** | **Count** | | **Proportion (%)** | |
| --- | --- | --- | --- | --- |
|  | **AM (CK)** | **AM-Res (Treatment)** | **AM (CK)** | **AM-Res (Treatment)** |
| **0-1** | 4128 | 4717 | 28.29 | 32.33 |
| **1-3** | 1558 | 1762 | 10.68 | 12.08 |
| **3-15** | 3306 | 3450 | 22.66 | 23.64 |
| **15-60** | 3298 | 2743 | 22.60 | 18.80 |
| **>60** | 2302 | 1920 | 15.78 | 13.16 |

Note: RPKM, Reads Per Kilo bases per Million reads. The number of reference genes is 13875.
