# Supplementary material for: Deep sequencing analysis of transcriptomes in Aspergillus flavus in response to resveratrol
Source: BMC Microbiol. 2015 Sep 16;15:182. doi: 10.1186/s12866-015-0513-6 (PMC4589122; doi:10.1186/s12866-015-0513-6)
Supplement: Additional file 6: — Histogram presentation of Gene Ontology (GO) enrichment analysis of differentially expressed genes. The x-axis indicates the number of genes in a GO term; * indicates the significantly differentially expressed GO term. (DOC 367 kb) [file 12866_2015_513_MOESM6_ESM.doc]

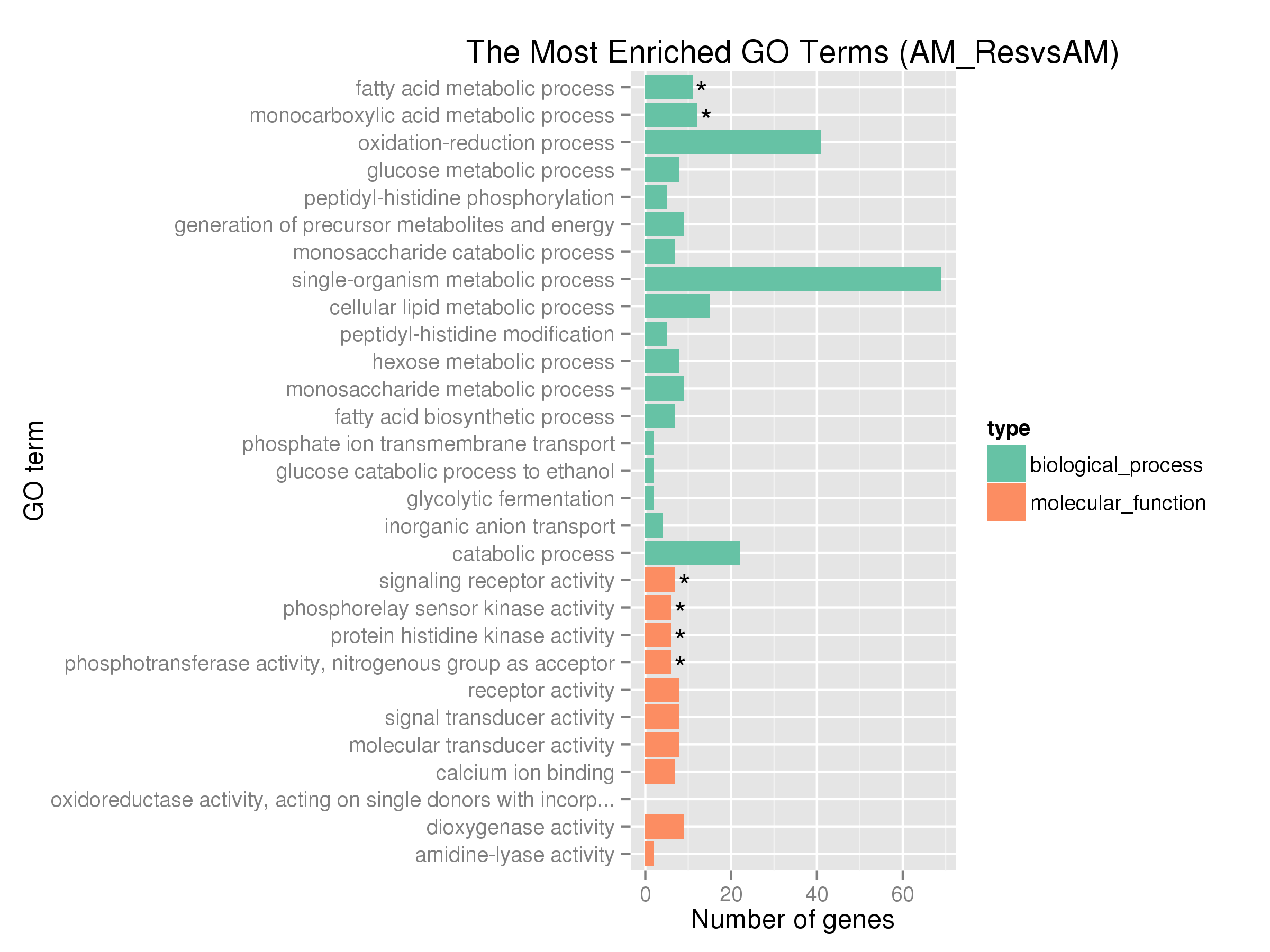


**Additional file 6** - Histogram presentation of GO enrichment analysis of differentially expressed genes. The x-axis indicates the number of genes in a GO term. * indicates the significantly differently expressed GO term.
