# Supplementary material for: Deep sequencing analysis of transcriptomes in Aspergillus flavus in response to resveratrol
Source: BMC Microbiol. 2015 Sep 16;15:182. doi: 10.1186/s12866-015-0513-6 (PMC4589122; doi:10.1186/s12866-015-0513-6)
Supplement: Additional file 8: — The dynamic change of aflatoxin content in the mediun with and without resveratrol. AM-Res (Treatment): A. flavus was cultured in A&M medium with resveratrol; AM (Control): A. flavus was cultured in A&M medium without resveratrol. (DOCX 14 kb) [file 12866_2015_513_MOESM8_ESM.docx]

**Additional file 8 - The dynamic change of aflatoxin content in the mediun with and without resveratrol**

AM (Control): *A. flavus* was cultured in A&M medium without resveratrol; AM-Res (Treatment): *A. flavus* was cultured in A&M medium with resveratrol.
